# Artificial Intelligence‐Driven Precision Bisphosphonate Therapy in Osteoporosis: Mechanisms, Clinical Applications, Risks, and Future Directions

**DOI:** 10.1111/os.70429

**Published:** 2026-09-17

**Authors:** Xiang Yao, Yicong Chao, Xiao Gu, Jingtao Peng, Jishan Yuan, Wei Shi, Fuguo Zhang, Qiwen Bao

**Affiliations:** ^1^ The Affiliated People's Hospital of Jiangsu University Zhenjiang Jiangsu Province China; ^2^ Jiangsu University Zhenjiang Jiangsu Province China; ^3^ Taizhou First People's Hospital Taizhou Zhejiang Province China

**Keywords:** artificial intelligence, bisphosphonates, deep learning, opportunistic screening, osteoporosis, precision medicine

## Abstract

Bisphosphonates remain the pharmacological standard of care for osteoporosis; however, their clinical efficacy is limited by significant diagnostic gaps, heterogeneous therapeutic responses, and persistent concerns regarding long‐term safety, including medication‐related osteonecrosis of the jaw (MRONJ) and atypical femoral fractures (AFF). To address these limitations, a structured comprehensive search of the Web of Science Core Collection, Scopus, and PubMed (January 2005–December 2025) was conducted to evaluate the intersection of bisphosphonate pharmacotherapy and computational medicine. The integration of artificial intelligence (AI) facilitates a methodological transition from empirical prescription to precision medicine across critical clinical domains. (1) Screening and Diagnosis: AI‐driven opportunistic screening utilizes routine computed tomography (CT) scans to automate asymptomatic vertebral fracture detection and assess bone quality via radiomics. (2) Treatment Stratification: Machine learning algorithms enable the prediction of individual bone mineral density (BMD) responses and optimize alternative or sequential therapies, facilitating precise patient selection for potent agents like zoledronic acid. (3) Safety Monitoring: Predictive models integrating clinical and imaging data allow for the pre‐emptive risk stratification of adverse events, shifting management from passive surveillance to active prevention. (4) Smart Delivery Systems: Furthermore, computational modeling accelerates the design of stimuli‐responsive nanocarriers to improve bone targeting and reduce systemic toxicity. Despite current challenges regarding algorithmic interpretability and multi‐center validation, incorporating emerging technologies—such as large language models (LLMs) for clinical triage and the integration of systemic metabolic networks (e.g., the gut‐bone axis)—suggests a promising trend toward a data‐informed, risk‐adjusted, and biologically targeted precision ecosystem in osteoporosis management.

## Graphical abstract

This comprehensive review integrates 17 benchmark studies to illustrate the transition of osteoporosis management toward precision medicine. The schematic highlights three key clinical applications: AI‐driven opportunistic screening from routine CTs, precision pharmacotherapy selection, and the predictive prevention of adverse events like MRONJ.

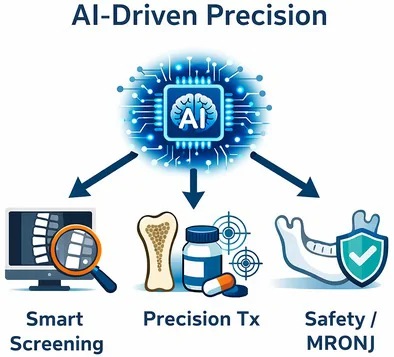

## 1 Introduction

Osteoporosis is a systemic skeletal disorder characterized by reduced bone mass and the microarchitectural deterioration of bone tissue, ultimately leading to an increased risk of fragility fractures. For over three decades, bisphosphonates (BPs) have served as the pharmacological cornerstone for managing this condition. BPs function by binding with high affinity to bone hydroxyapatite and inhibiting farnesyl pyrophosphate synthase within osteoclasts, thereby effectively suppressing bone resorption [1, 2]. Despite their proven efficacy in reducing vertebral, non‐vertebral, and hip fractures, the global burden of osteoporosis remains staggering, causing significant morbidity, mortality, and economic strain on healthcare systems worldwide [3, 4]. Furthermore, clinical practice is increasingly challenged by the inherent drawbacks of the traditional “one‐size‐fits‐all” treatment strategy. Patient responses to BPs are highly heterogeneous, and suboptimal long‐term adherence (often < 50% at 1 year) contributes to a widening “treatment gap” where high‐risk individuals remain unprotected [5, 6, 7]. Prolonged suppression of bone turnover also raises serious clinical concerns regarding rare but devastating adverse events, such as medication‐related osteonecrosis of the jaw (MRONJ) and atypical femoral fractures (AFF) [8, 9]. Additionally, standard diagnostic tools like dual‐energy x‐ray absorptiometry (DXA) often fail to capture microstructural deterioration in complex populations, particularly in those with type 2 diabetes mellitus (T2DM) and age‐related systemic alterations, where patients sustain fractures despite having normal bone mineral density (the “bone paradox”) [10, 11].

To address these limitations, the integration of Artificial Intelligence (AI) into metabolic bone disease management offers a highly practical alternative to conventional empirical approaches. AI broadly encompasses computational systems designed to perform tasks that typically require human intelligence. Within this field, Machine Learning (ML) utilizes statistical algorithms to identify patterns in data, while Deep Learning (DL) employs multi‐layered artificial neural networks to analyze highly complex, high‐dimensional datasets, such as medical imaging and genomic profiles [12, 13]. In the context of osteoporosis and BP therapy, AI presents significant clinical opportunities: it enables opportunistic screening through automated CT analysis to identify candidates earlier [14], utilizes radiomics to quantify “invisible” trabecular quality defects [15], and powers predictive algorithms to forecast individual drug responses and safety risks before therapy initiation [16]. However, the application of AI in this field also faces notable challenges, including the “black‐box” nature of deep learning algorithms lacking clinical interpretability, the necessity for robust multi‐center prospective validation, and strict data privacy concerns.

Moving beyond the well‐established general pharmacology of BPs, the present review focuses specifically on the intersection of AI and precision pharmacotherapy. Emerging evidence is critically synthesized regarding how AI‐driven strategies are refining risk stratification, optimizing sequential therapy, and enabling intelligent safety monitoring. To the best of the authors' knowledge, this is the first comprehensive review to comprehensively evaluate the transition from empirical bisphosphonate prescription to data‐driven precision medicine. By detailing this methodological transition, this study aims to provide a roadmap for the next generation of safe and individualized anti‐osteoporosis therapy. A detailed comparison between traditional empirical management and the proposed AI‐driven precision framework is provided in Table 1.

**TABLE 1:** Comparison between traditional empirical management and AI‐driven precision management of bisphosphonates.

Clinical domain	Traditional management (empirical)	AI‐driven precision management	Key AI technologies
Screening strategy	Passive: Relies on DXA referrals; > 70% high‐risk patients are missed (diagnosis gap).	Proactive (Opportunistic): Automated analysis of routine CT scans (Chest/Abdomen) performed for other indications.	Deep learning (CNNs) for vertebral segmentation and fracture detection.
Diagnosis & bone quality	Density‐based: Relies on T‐scores (DXA). Misses fracture risk in T2DM (Bone Paradox).	Quality‐Based: Analyzes trabecular microarchitecture and texture patterns to identify “fragile bone quality” independent of density.	Radiomics, texture analysis, finite element analysis (FEA).
Therapeutic decision	Trial‐and‐error: “One‐size‐fits‐all” prescription. Response assessed only after 1–2 years.	Predictive stratification: Predicts individual BMD response and fracture reduction probability before initiation.	Machine learning (ML) algorithms (e.g., Random Forest, Gradient Boosting).
Safety monitoring (ONJ/AFF)	Generic avoidance: broad warnings (e.g., “avoid dental extraction”).	Risk Scoring: Calculates personalized probability scores based on cumulative dose, extraction history, and co‐medications.	Data mining, NLP on electronic health records (EHR).
Drug delivery	Systemic distribution: low oral bioavailability (< 1%); systemic toxicity.	Smart Targeting: Stimuli‐responsive carriers release drug only at active resorption sites (acidic microenvironment).	Computational chemistry for nanocarrier design.

## 2 Methods

### 2.1 Search Strategy and Selection Criteria

A comprehensive search of the Web of Science Core Collection, Scopus, and PubMed databases was conducted for literature published from January 1, 2005, through December 31, 2025. The search strategy employed a logic‐grid approach combining three concept clusters: (1) Osteoporosis and Fragility Fractures, (2) Artificial Intelligence and Machine Learning, and (3) Bisphosphonates and Precision Medicine. Specific search terms included “Osteoporosis,” “Bisphosphonates,” “Deep Learning,” “Opportunistic Screening,” and “Adverse Event Prediction” (see Table S1 for full search strings).

### 2.2 Data Extraction and Study Selection

**FIGURE 1:**
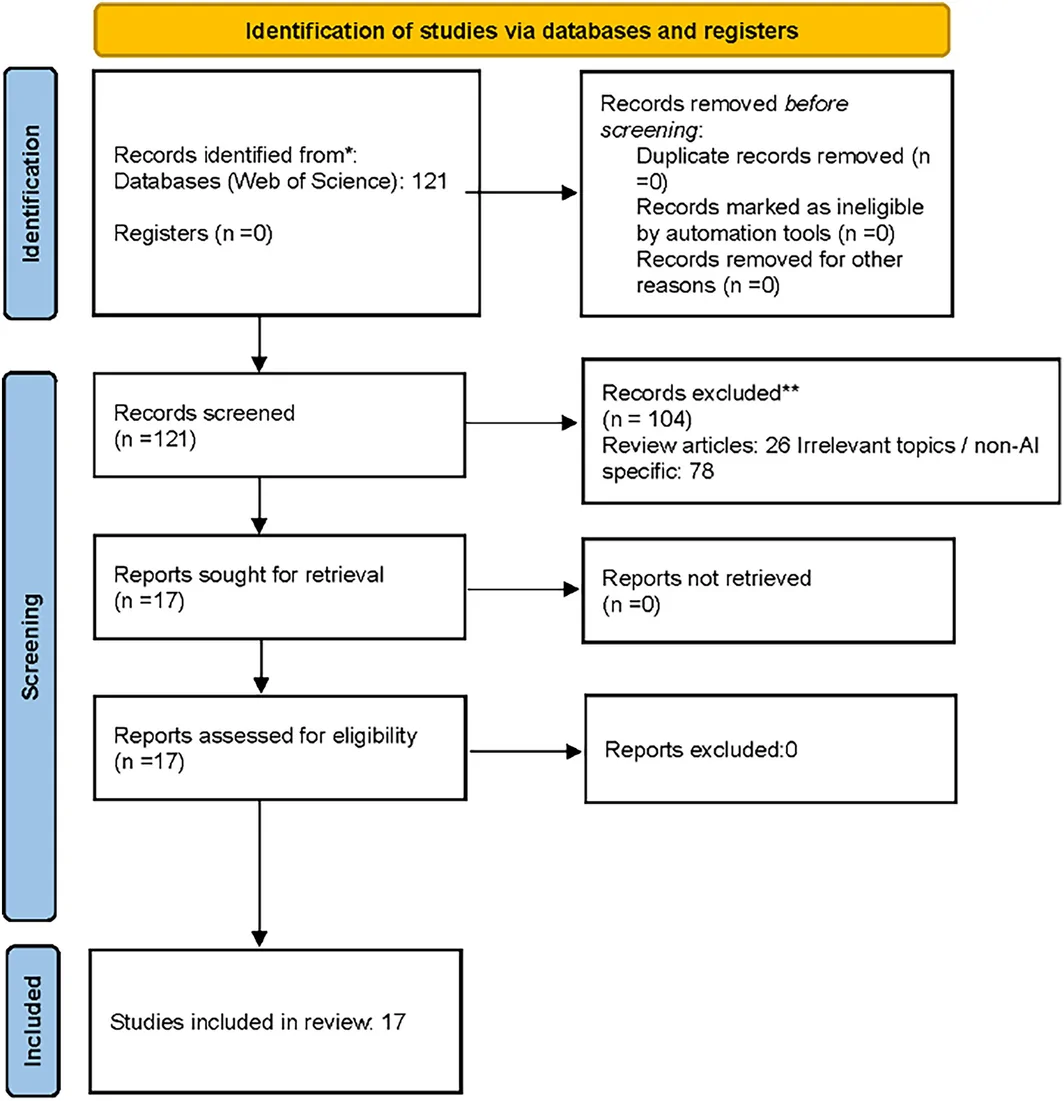
PRISMA 2020 flow diagram illustrating the selection process. The initial search identified 121 records. After excluding review articles (*n* = 26) and non‐relevant or non‐AI‐specific studies (*n* = 78), 17 original research articles were included for the final synthesis. *Consider, if feasible to do so, reporting the number of records identified from each database or register searched (rather than the total number across all databases/registers). **If automation tools were used, indicate how many records were excluded by a human and how many were excluded by automation tools. *Source:* Page MJ, et al. *BMJ* 2021;372:n71. https://doi.org/10.1136/bmj.n71. This work is licensed under CC BY 4.0. To view a copy of this license, visit: https://creativecommons.org/licenses/by/4.0/ .

The initial search yielded 121 potentially relevant records across the selected databases. Reference management and duplicate removal were performed using EndNote 20 (Clarivate Analytics, Philadelphia, PA, USA). To ensure the rigor and relevance of this review, priority was given to original research articles that provided primary data on AI model performance or clinical validation. Review articles (*n* = 26) and studies lacking full datasets were excluded from the primary synthesis, although select key reviews were consulted for background context. After screening titles and abstracts, 42 original research articles identified through this comprehensive search were included in the final qualitative synthesis. The detailed selection process is illustrated in the PRISMA flow diagram (Figure 1).

### 2.3 Quality Assessment

Although this study is structured as a comprehensive narrative review rather than a formal systematic meta‐analysis, a foundational quality assessment of the 17 included original research articles was conducted to ensure the reliability of the synthesized evidence. The methodological quality of diagnostic and predictive AI models was evaluated using principles derived from the Quality Assessment of Diagnostic Accuracy Studies (QUADAS‐2) tool and the modified Newcastle‐Ottawa Scale (NOS) for observational cohorts. Overall, the assessed studies demonstrated moderate‐to‐high quality in algorithm training and internal validation. However, common methodological limitations were observed, most notably a prevalent reliance on retrospective, single‐center datasets and a general lack of external, multi‐center prospective validation cohorts, which introduces potential risks of spectrum bias and limits broad clinical generalizability.

## 3 AI‐Driven Opportunistic Screening and Diagnosis

Although DXA remains the clinical gold standard for diagnosis, its widespread application is hindered by significant underutilization and technical limitations. Degenerative changes, such as aortic calcification or osteophytes, can artificially elevate BMD readings, leading to false‐negative diagnoses in older populations [17, 18]. Consequently, a widespread “diagnosis gap” persists; it is estimated that over 70% of high‐risk patients who sustain a fragility fracture have never undergone DXA screening [8]. To bridge this gap, AI facilitates opportunistic screening—the automated analysis of routine computed tomography (CT) scans performed for other clinical indications (e.g., chest CT for lung cancer screening or abdominal CT for gastrointestinal evaluation). Recent studies confirm that CT‐derived Hounsfield Units (HU) correlate strongly with DXA T‐scores, enabling AI algorithms to assess bone health without additional radiation exposure or diagnostic costs [19, 20].

### 3.1 Automated Vertebral Fracture Detection

Vertebral compression fractures (VCFs) are strong predictors of future hip fractures; yet nearly two‐thirds remain asymptomatic and are frequently unreported in routine radiological workflows. Retrospective studies indicate that up to 50% of incidental VCFs visible on chest or abdominal CTs are not documented in final radiology reports, representing a critical missed opportunity for secondary prevention [21]. Deep learning algorithms, specifically Convolutional Neural Networks (CNNs), demonstrate diagnostic accuracy comparable to expert radiologists in identifying these subtle fractures. Recent applications of DL models to routine chest CTs have achieved high sensitivity in detecting osteoporotic fractures, surpassing routine manual reporting by flagging asymptomatic fractures that prompt bisphosphonate intervention [14, 22]. Integrating these algorithms into hospital picture archiving and communication systems (PACS) allows for the automated identification of therapeutic candidates who might otherwise be overlooked.

### 3.2 CT‐Based Bone Mineral Density (BMD) Assessment

Beyond fracture detection, AI enables the quantitative extraction of volumetric BMD from non‐calibrated CT scans. By training on paired CT and DXA datasets, “asynchronous calibration” algorithms can convert HU into reliable T‐scores [14, 23]. A 2025 study by Wu et al. demonstrated that AI‐assisted automatic screening using chest CT images showed a strong correlation with central DXA and effectively stratified fracture risk across different scanner manufacturers [14]. This technology repurposes existing archival CT scans into opportunistic diagnostic tools, identifying a vast pool of untreated patients eligible for bisphosphonate initiation [24].

### 3.3 Beyond Density: Radiomics and Bone Quality

Traditional DXA measures areal bone density and often fail to predict fractures in conditions such as T2DM, where bone density may appear normal while bone quality is compromised. Radiomics—a computational method that extracts high‐dimensional texture features from medical images—provides a robust alternative approach. Machine learning models combining quantitative CT (QCT) and finite element analysis (FEA) can identify subtle microstructural deterioration, including increased cortical porosity and trabecular dysconnectivity [15]. These algorithm‐derived biomarkers provide a more precise indication for bisphosphonate therapy than traditional T‐scores alone, particularly in complex patient populations exhibiting the “bone paradox” [25].

## 4 AI‐Driven Precision Treatment Stratification and Response Prediction

**FIGURE 2:**
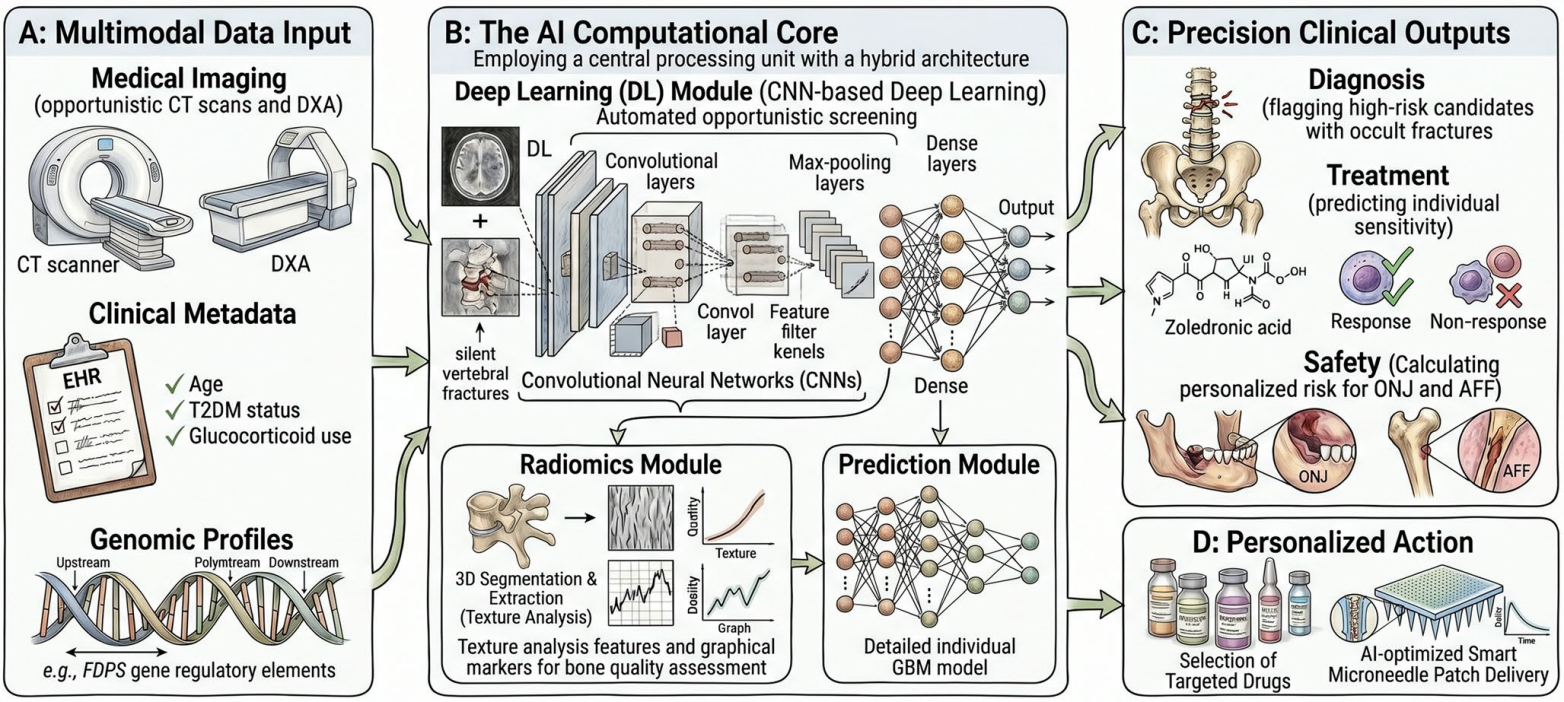
The Al‐augmented precision care pathway for bisphosphonate therapy. This schematic framework illustrates the transformation of osteoporosis management from empirical prescription to a data‐driven precision ecosystem. (A) Multimodal Data Input: The pathway integrates high‐dimensional heterogeneous data, aggregating medical imaging (opportunistic CT scans and DXA), clinical metadata from Electronic Health Records (e.g., age, T2DM status, glucocorticoid use), and genomic profiles (e.g., FDPS polymorphisms). (B) The AI Computational Core: The central processing unit employs a hybrid architecture. Deep Learning (DL) modules, utilizing Convolutional Neural Networks (CNNs), perform automated opportunistic screening to detect silent vertebral fractures. Simultaneously, the Radiomics Module extracts texture analysis features to assess bone quality, while the Prediction Module uses Machine Learning (e.g., GBM) to integrate clinical variables. (C) Precision clinical outputs: The processed data translates into three actionable insights: (1) Diagnosis, flagging high‐risk candidates with occult fractures; (2) Treatment, predicting individual therapeutic sensitivity (response vs. non‐response) to zoledronic acid; and (3) Safety, calculating a personalized risk score for adverse events such as ONJ and AFF. (D) Personalized Action: These outputs guide the final clinical decision, facilitating the selection of targeted drugs or AI‐optimized smart delivery systems tailored to the patient's specific risk profile. AFF, atypical femoral fracture; CNN, convolutional neural network; DXA, dual‐energy x‐ray absorptiometry; GBM, gradient boosting machine; ONJ, osteonecrosis of the jaw; T2DM, type 2 diabetes mellitus. This figure was drafted with the assistance of Grok (xAI, Burlingame, CA, USA) and manually revised by the authors.

Following the identification of high‐risk patients, the clinical challenge shifts to selecting the optimal therapeutic strategy [16]. The historical “treat‐to‐target” approach often relies on an empirical, trial‐and‐error methodology. The application of Artificial Intelligence facilitates a transition from reactive monitoring to predictive precision medicine. A comprehensive AI‐augmented precision care pathway is proposed, integrating multimodal data input with deep learning processing to guide clinical decision‐making, as illustrated in Figure 2.

### 4.1 Predicting Therapeutic Response: The Case of Zoledronic Acid

Zoledronic acid (ZA), administered as a once‐yearly infusion, requires careful patient selection due to its long skeletal retention. Pivotal trials, such as the HORIZON‐Pivotal Fracture Trial, have established ZA as a standard of care for reducing hip and vertebral fractures [26, 27]. However, approximately 15%–30% of patients fail to achieve expected BMD gains due to genetic or metabolic heterogeneity [28]. Machine learning (ML) models offer predictive capabilities that transition clinical management from reactive measurement to proactive prediction. Recent studies utilizing ML algorithms—integrating baseline age, BMI, bone turnover markers, and radiomic features—have achieved > 80% accuracy in predicting individual BMD responses to bisphosphonates [16]. Consequently, for patients predicted to exhibit a suboptimal response to ZA, AI‐supported clinical decision systems can suggest alternative therapies avoiding prolonged periods of ineffective treatment [12].

### 4.2 Predicting Outcomes for Alternative and Sequential Therapies

Beyond bisphosphonates, AI algorithms are increasingly applied to optimize the selection and sequencing of other advanced anti‐osteoporosis medications, including anabolic agents (e.g., teriparatide and romosozumab) and potent antiresorptives (e.g., denosumab). Because these agents have distinct mechanisms of action and varying rebound risks upon discontinuation, AI modeling assists in formulating optimal sequential therapy strategies. For instance, ML models analyzing baseline bone turnover markers and structural geometry can predict the precise magnitude of BMD response to teriparatide, allowing clinicians to identify the most suitable candidates for anabolic intervention. Furthermore, predictive algorithms are being developed to determine the optimal timing for transitioning patients from romosozumab or denosumab back to bisphosphonates to effectively consolidate bone density gains and prevent the well‐documented rebound vertebral fracture phenomenon.

### 4.3 AI In Glucocorticoid‐Induced Osteoporosis (GIOP) Management

GIOP is the most common form of secondary osteoporosis; however, clinical implementation frequently remains inconsistent with established guidelines [29]. Despite clear evidence that bisphosphonates prevent bone loss in GIOP [30, 31], widespread clinical inertia contributes to significant undertreatment. AI‐driven Clinical Decision Support Systems (CDSS) provide a mechanism to address this gap. By utilizing natural language processing (NLP) to mine Electronic Health Records (EHR), AI systems can automatically calculate a patient's cumulative glucocorticoid exposure and flag those meeting intervention criteria. Studies have demonstrated that AI‐triggered alert systems significantly increase bisphosphonate prescription rates in high‐risk GIOP patients by over 30% [32], effectively translating guideline recommendations into clinical protection.

### 4.4 Tackling the “Bone Paradox” in Type 2 Diabetes (T2DM)

Patients with T2DM frequently present with normal or elevated BMD yet experience high fracture risk—a phenomenon termed the “bone paradox,” primarily driven by the accumulation of advanced glycation end‐products (AGEs) and microstructural deterioration [33]. AI models integrating radiomics and clinical metadata (e.g., HbA1c variability) have outperformed standard BMD metrics in predicting fractures within diabetic cohorts [25]. By analyzing trabecular microarchitecture patterns and extracting high‐dimensional radiomic features (e.g., Gray Level Co‐occurrence Matrix [GLCM] entropy and skewness) from opportunistic CT scans, these algorithms identify diabetic patients with compromised bone quality who require pharmacological intervention despite presenting with non‐osteoporotic T‐scores, thereby expanding therapeutic indications to a vulnerable population [15, 25].

## 5 AI‐Enhanced Safety Monitoring and Smart Delivery Systems

The clinical utility of potent bisphosphonates, such as intravenous zoledronic acid, is frequently limited by patient concerns regarding rare but severe adverse events. Medication‐related osteonecrosis of the jaw (MRONJ) and atypical femoral fractures (AFF) have emerged as dominant clinical priorities in recent bibliometric analyses [34, 35]. Concerns over these complications contribute significantly to suboptimal treatment adherence. The application of artificial intelligence facilitates a transition from passive clinical surveillance to predictive risk stratification and targeted intervention.

### 5.1 AI‐Driven Prediction of ONJ

MRONJ represents a significant complication, particularly in oncology cohorts receiving high‐dose bisphosphonate therapy [34]. Traditional risk management relies on the generalized avoidance of invasive dental procedures, a strategy lacking individual specificity. Recent computational advancements utilize machine learning (ML) to stratify risk prior to the onset of adverse events [36]. For example, gradient boosting machine (GBM) algorithms trained on longitudinal patient data—integrating variables such as cumulative bisphosphonate dose, dental extraction history, and corticosteroid co‐prescription—have demonstrated high sensitivity in predicting MRONJ occurrence [37]. Furthermore, data mining applications analyzing the FDA Adverse Event Reporting System (FAERS) have generated nomograms that quantify the synergistic risks of bisphosphonates when co‐administered with specific angiogenic inhibitors [36]. These predictive tools generate personalized safety scores, guiding targeted dental clearance for high‐risk individuals while minimizing unnecessary treatment barriers for the low‐risk majority.

### 5.2 Early Detection of Atypical Femoral Fractures (AFF)

AFFs are stress fractures associated with the prolonged suppression of bone turnover [38]. Diagnosis is frequently delayed because prodromal pain and subtle radiographic alterations are easily overlooked in routine clinical practice. Deep learning models are increasingly applied to enhance AFF screening. CNNs trained on femoral radiographs can identify subtle hallmarks of AFF, including focal lateral cortical thickening and transverse fracture lines (“beaking”) [14, 35, 39]. By screening archival images to flag pre‐AFF features, these algorithms can prompt timely cessation of therapy in asymptomatic patients. Additionally, AI algorithms are being evaluated to analyze longitudinal trajectories of bone turnover markers (e.g., CTX and P1NP) to identify over‐suppression profiles, thereby optimizing the timing of drug holidays as recommended in recent clinical guidelines [40, 41].

### 5.3 Intelligent Targeted Drug Delivery Systems

To further mitigate systemic side effects and address the low oral bioavailability of traditional bisphosphonates, computational modeling is accelerating the design of stimuli‐responsive, bone‐targeting nanocarriers. These intelligent delivery systems utilize bisphosphonate‐modified surfaces to achieve high affinity for bone hydroxyapatite, releasing therapeutic payloads specifically within the acidic microenvironment of active resorption sites. Furthermore, emerging evidence suggests that the efficacy and safety of such targeted interventions may be modulated by systemic metabolic factors, including the state of the gut microbiota and the gut‐bone axis [13]. Integrating microbiome signatures into AI models represents a promising frontier for optimizing these advanced delivery systems and ensuring individualized safety. To illustrate the practical translation of these computational technologies, a curated selection of highly representative benchmark studies—highlighting distinct AI applications across screening, diagnosis, and safety monitoring—is summarized in Table 2.

**TABLE 2:** Key studies utilizing artificial intelligence in bisphosphonate management.

Study (author, year)	Clinical application	Application AI methodology/algorithm	Data source	Key outcome/performance
Wu et al. (2025)	Opportunistic screening	Deep Learning (CNNs)	Chest CT scans (*n* = 2482)	Automated BMD assessment achieved high correlation (*r* = 0.87) with DXA T‐scores across different scanners.
Liu et al. (2025)	Bone quality assessment	Radiomics + machine learning	QCT/Finite element analysis	Radiomic features successfully predicted femoral strength and fracture risk better than density alone.
Tanphiriyakun et al. (2021)	Therapeutic response	Gradient boosting machine (GBM)	Clinical baseline data + biomarkers	Predicted individual BMD response with 82.3% accuracy, identifying non‐responders prior to treatment.
Morikawa T, et al. (2022)	GIOP Management	Natural language processing (NLP)	Electronic health records (EHR)	Automated alerts increased bisphosphonate prescription rates in high‐risk steroid users by 34%.
Kim et al. (2018)	Safety (ONJ prediction)	Machine	Longitudinal claims data	Developed a risk scoring model integrating dose, duration, and extraction history (AUC = 0.81).
Wei et al. (2024)	Pharmacovigilance	Data mining algorithms	FAERS database	Identified novel synergistic risks between zoledronic acid and angiogenic inhibitors.

## 6 Opportunities, Challenges, and Clinical Realities

The integration of AI into osteoporosis management presents significant opportunities—such as bridging the diagnostic gap and individualizing pharmacotherapy—alongside notable technical and clinical challenges.

### 6.1 Data Quality and Algorithmic Bias

AI models are fundamentally dependent on the quality and representativeness of their training datasets [42]. Currently, the majority of algorithms are trained on retrospective, single‐center datasets that frequently lack demographic diversity. For instance, if a fracture prediction model is trained predominantly on data from Caucasian postmenopausal women, its predictive accuracy may decrease significantly when applied to male patients or diverse ethnic groups, potentially exacerbating existing healthcare disparities [42, 43].

### 6.2 The “Black Box” Problem

A primary barrier to widespread clinical adoption is the lack of interpretability inherent in complex deep learning architectures. Clinicians often hesitate to rely on therapeutic recommendations—such as discontinuing a drug due to a predicted AFF risk—derived from opaque computational processes [44]. The implementation of Explainable AI (XAI) methodologies is essential to foster clinical trust. Techniques such as case‐based reasoning (CBR) and Gradient‐weighted Class Activation Mapping (Grad‐CAM), which visually highlight the specific radiological features (e.g., cortical texture alterations) driving the algorithm's predictions, are critical for facilitating transparent decision‐making [42].

### 6.3 Lack of Prospective Validation and Clinical Accountability

At present, much of the evidence supporting AI in osteoporosis management is derived from retrospective, in silico validation. There remains a substantial gap regarding prospective, randomized controlled trials (RCTs) demonstrating that AI‐guided bisphosphonate management definitively improves patient outcomes—such as reducing fracture rates or MRONJ incidence—compared to standard guideline‐based care [28]. Until robust Level I evidence is established, AI tools must be conceptualized as clinical decision‐support aids rather than autonomous prescribers [12]. The legal and ethical responsibility for final therapeutic decisions rests with the clinician, necessitating a “human‐in‐the‐loop” workflow to mitigate algorithmic errors and ensure patient safety [42, 45].

### 6.4 Modeling Systemic Complexity and the Gut‐Bone Axis

A further challenge lies in the multidimensional nature of bone metabolism. Current AI models predominantly rely on localized imaging or standard clinical variables. However, emerging research emphasizes that osteoporosis is a systemic manifestation of biological aging, significantly influenced by extra‐skeletal networks such as the gut microbiota. Understanding and incorporating the complex interplay of the gut‐bone axis into predictive AI models remains a critical frontier for accurately capturing metabolic heterogeneity and advancing comprehensive anti‐aging skeletal therapies [46].

## 7 Future Perspectives: From Algorithms to Actionable Care

While artificial intelligence demonstrates robust diagnostic capabilities, future advancements depend on integrating predictive models with actionable clinical interventions. The convergence of large language models (LLMs), advanced drug delivery systems, and multi‐omics profiling offers a pathway to transition from static drug regimens to dynamic, individualized therapeutic strategies.

### 7.1 Generative AI and Large Language Models (LLMs)

The integration of LLMs presents novel solutions for optimizing clinical workflows and patient adherence. Beyond functioning as advanced Clinical Decision Support Systems (CDSS) that interpret opaque algorithm predictions to enhance physician trust [42], LLMs are demonstrating significant utility in administrative and logistical prioritization. A recent pilot study highlighted the clinical utility of LLMs for osteoporosis referral triage, demonstrating that these models can effectively assist in the initial assessment and prioritization of patients within specialized clinics [47]. Such applications may alleviate the burden on orthopedic surgeons by automating routine triage tasks. Concurrently, patient‐facing LLM interfaces can explain complex risks (e.g., MRONJ) in accessible language and provide tailored adherence reminders based on individual behavioral patterns [12, 14].

### 7.2 AI‐Optimized Smart Drug Delivery Systems

A critical application of computational modeling lies in the design of targeted delivery systems. Machine learning algorithms are utilized to simulate molecular interactions, accelerating the discovery of novel bisphosphonate‐functionalized carriers that improve bone affinity while reducing systemic toxicity.

**FIGURE 3:**
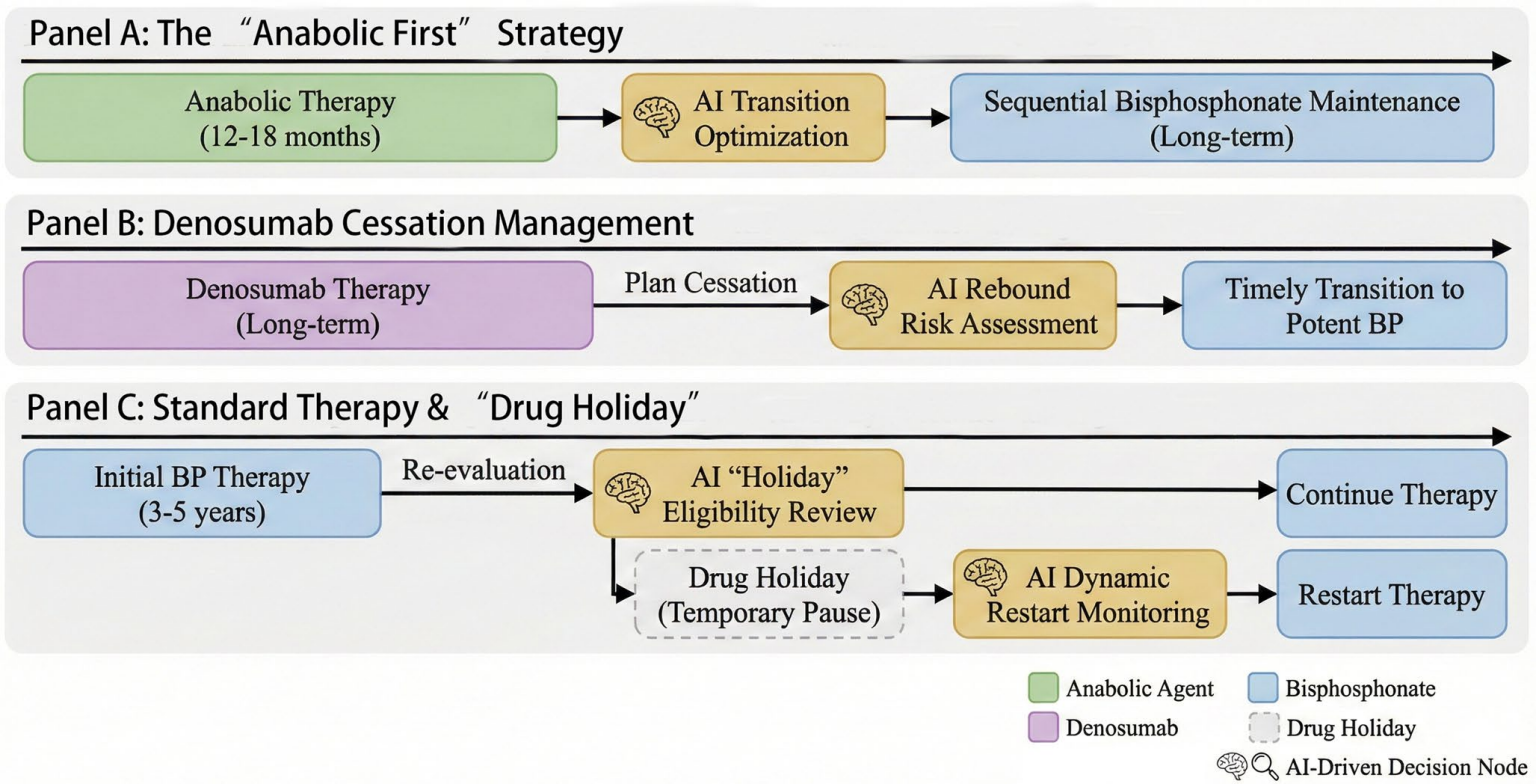
AI‐optimized smart drug delivery systems for precision bisphosphonate therapy. This schematic illustrates the synergy between computational intelligence and material innovation in modernizing bisphosphonate pharmacotherapy. 1. Design Phase (Left): Machine learning algorithms are utilized to perform high‐throughput molecular interactions and docking simulations. This AI‐guided design accelerates the discovery of nanoparticle carriers with optimized physicochemical properties and superior bone‐mineral affinity. 2. Circulation (Middle): Engineered smart delivery systems, such as PEGylated nanoparticles or targeted conjugates, demonstrate enhanced stability within the systemic circulation. By minimizing off‐target interactions with healthy tissues and reducing renal clearance, these systems significantly mitigate systemic toxicity and potential side effects 3. Targeting & Release (Right): The therapeutic efficacy is maximized through stimuli‐responsive release mechanisms. Upon reaching active bone resorption pits, the nanoparticle's sensitive “shell” responds specifically to the localized acidic microenvironment (decreased pH) created by osteoclast activity. This trigger ensures that bisphosphonates are released exclusively at sites of active skeletal deterioration, effectively concentrating the drug where it is most needed. AI, artificial intelligence; pH, potential of hydrogen (acidic microenvironment); PEG, polyethylene glycol.

1. Nanoparticle Carriers: AI models assist in optimizing the physicochemical properties of liposomes and ionizable lipid nanoparticles to enhance targeted delivery to active resorption sites [48, 49, 50].
2. Targeted Conjugates: Computational design facilitates the development of conjugates, such as PEGylated alendronate or hydroxyapatite‐binding motifs, which minimize renal clearance and extend drug half‐life [51, 52].
3. Stimuli‐Responsive Hydrogels: AI‐guided material science is advancing hydrogel systems designed to release therapeutic payloads exclusively in response to the acidic microenvironment (pH < 6.5) characteristic of osteoclast activity [36, 53]. This mechanism is schematically depicted in Figure 3.

These innovations, guided by computational prediction, represent the material foundation of future precision therapy. AI prediction models will play a crucial role in pharmacoeconomics, identifying which high‐risk patient subgroups justify the cost of these advanced delivery systems versus standard generic formulations [54].

### 7.3 Digital Twins and Multimodal Integration

Current predictive models predominantly rely on clinical and radiological modalities. Future multimodal AI systems will likely integrate systemic multi‐omics profiles to construct comprehensive “Digital Twins” virtual replicas of a patient's skeletal and metabolic physiology. Emerging evidence indicates that dietary patterns and gut microbiota‐related metabolites represent an inextricable link in bone metabolism, significantly influencing both fracture risk and therapeutic outcomes [55]. Future precision medicine platforms may incorporate microbiome signatures alongside genomic data (e.g., FDPS polymorphisms) and real‐time biomechanical inputs from wearable biosensors [25, 28]. This comprehensive integration will allow clinicians to identify novel therapeutic targets across the gut‐bone axis [11] and simulate the long‐term effects of various bisphosphonate regimens on the digital twin before initiating actual pharmacotherapy.

## 8 Limitations of the Review

While this review comprehensively maps the intersection of artificial intelligence and bisphosphonate therapy, several limitations must be acknowledged. First, as a comprehensive narrative review rather than a quantitative meta‐analysis, the literature selection and synthesis are inherently susceptible to subjective selection bias. Second, the vast heterogeneity in AI methodologies, ranging from CNNs for imaging to NLP for electronic health records, precludes standardized performance comparisons across studies. Finally, the majority of the cited literature relies on retrospective, single‐center datasets. The rapid evolution of AI models often outpaces the execution of robust, prospective RCTs. Consequently, the predictive efficacies summarized herein predominantly reflect in silico performance rather than real‐world clinical outcomes.

## 9 Conclusion

For over three decades, bisphosphonates have stood as the pharmacological backbone of osteoporosis management, supported by robust evidence from pivotal trials [26, 27]. However, traditional clinical models, which are often empirical and generalized, are increasingly limited by vast diagnostic gaps, heterogeneous treatment responses, and persistent concerns regarding long‐term safety [29]. The integration of artificial intelligence facilitates a methodological transition toward precision pharmacotherapy. The application of AI‐driven opportunistic screening repurposes routine CT scans to identify asymptomatic high‐risk populations [14]. Concurrently, radiomics and machine learning methodologies help address the “bone paradox” in complex conditions such as Type 2 Diabetes [25] and enable the prediction of individual therapeutic responses prior to drug administration [16]. Furthermore, the management of rare adverse events, including MRONJ and AFF, is evolving from passive clinical avoidance to data‐driven, precise risk stratification [36, 37].

Looking forward, future research directions must focus on translating these computational models into actionable clinical applications and therapeutic development. The synergy between machine learning and material innovation will be critical in optimizing smart, stimuli‐responsive drug delivery systems to minimize toxicity and overcome adherence barriers [48, 49]. While critical challenges concerning algorithmic bias, model opacity, and the necessity for multi‐center prospective validation persist [42, 43], the incorporation of systemic physiological networks—such as the gut‐bone axis and comprehensive multi‐omics profiles—represents an essential frontier for future models. Ultimately, the convergence of these high‐dimensional data streams with advanced algorithms suggests a promising trend toward a precision medical ecosystem, ensuring that anti‐osteoporosis therapy becomes consistently data‐informed, risk‐adjusted, and biologically targeted.

## Author Contributions

**Xiang Yao:** conceptualization, methodology, supervision, funding acquisition, writing – review and editing. **Yicong Chao:** formal analysis, visualization. **Xiao Gu:** validation, writing – review and editing. **Jingtao Peng:** formal analysis, validation, writing – review and editing. **Jishan Yuan:** writing – original draft, software, visualization, data curation, investigation. **Wei Shi:** investigation, validation, writing – review and editing. **Fuguo Zhang:** writing – review and editing, validation, investigation. **Qiwen Bao:** writing – original draft, software, visualization, data curation, investigation.

## Funding

This work was supported by the Taizhou Municipal Science and Technology Project (Grant No. 22ywb40 and Grant No. 22ywa25).

## Ethics Statement

The authors have nothing to report.

## Conflicts of Interest

The authors declare no conflicts of interest.

## Supporting information


**Table S1:** Detailed search strategy and query strings.

## Data Availability

Data sharing not applicable to this article as no datasets were generated or analysed during the current study.
