# Supplementary material for: Artificial Intelligence‐Driven Precision Bisphosphonate Therapy in Osteoporosis: Mechanisms, Clinical Applications, Risks, and Future Directions
Source: Orthop Surg. 2026 Sep 17:10.1111/os.70429. Online ahead of print. doi: 10.1111/os.70429 (PMC13585479; doi:10.1111/os.70429)
Supplement: Supplementary file 1 [file OS-9999-0-s001.docx]

**Table S1. Detailed Search Strategy and Query Strings**

| Database | Search Query (Jan 2005 – Dec 2025) |
| --- | --- |
| PubMed | ("Osteoporosis"[MeSH] OR "Bone Density"[Title/Abstract]) AND ("Bisphosphonates"[MeSH] OR "Zoledronic Acid" OR "Alendronate") AND ("Artificial Intelligence"[MeSH] OR "Deep Learning" OR "Machine Learning" OR "Opportunistic Screening" OR "Radiomics") |
| Web of Science | TS=(Osteoporosis OR "Fragility Fracture") AND TS=(Bisphosphonate* OR "Zoledronic Acid") AND TS=("Artificial Intelligence" OR "Machine Learning" OR "Deep Learning" OR "Neural Network*" OR "Radiomics") |
| Scopus | TITLE-ABS-KEY ( ( osteoporosis OR "fragility fracture" ) AND ( bisphosphonate* ) AND ( "Artificial Intelligence" OR "Machine Learning" OR "Deep Learning" OR "Predictive Model" ) ) |
